# Heatmap-Based Active Shape Model for Landmark Detection in Lumbar X-ray Images

**DOI:** 10.1007/s10278-024-01210-x

**Published:** 2024-08-05

**Authors:** Minho Choi, Jun-Su Jang

**Affiliations:** Digital Health Research Division, Korea Institute of Oriental Medicine, 1672 Yuseong-daero, Yuseong-gu, Daejeon, 34054 Republic of Korea

**Keywords:** Lumbar X-ray image, Landmark correction, Active shape model, Heatmap response

## Abstract

Medical staff inspect lumbar X-ray images to diagnose lumbar spine diseases, and the analysis process is currently automated using deep-learning techniques. The detection of landmarks is necessary in the automatic process of localizing the position and identifying the morphological features of the vertebrae. However, detection errors may occur owing to the noise and ambiguity of images, as well as individual variations in the shape of the lumbar vertebrae. This study proposes a method to improve the robustness of landmark detection results. This method assumes that landmarks are detected by a convolutional neural network-based two-step model consisting of Pose-Net and M-Net. The model generates a heatmap response to indicate the probable landmark positions. The proposed method then corrects the landmark positions using the heatmap response and active shape model, which employs statistical information on the landmark distribution. Experiments were conducted using 3600 lumbar X-ray images, and the results showed that the landmark detection error was reduced by the proposed method. The average value of maximum errors decreased by 5.58% after applying the proposed method, which combines the outstanding image analysis capabilities of deep learning with statistical shape constraints on landmark distribution. The proposed method could also be easily integrated with other techniques to increase the robustness of landmark detection results such as CoordConv layers and non-directional part affinity field. This resulted in a further enhancement in the landmark detection performance. These advantages can improve the reliability of automatic systems used to inspect lumbar X-ray images. This will benefit both patients and medical staff by reducing medical expenses and increasing diagnostic efficiency.

**Supplementary Information:**

The online version contains supplementary material available at 10.1007/s10278-024-01210-x.

## Introduction

The lumbar vertebrae, which connect the thoracic vertebrae and sacrum, consist of five vertebrae, known as L1 to L5. The lumbar vertebrae are the largest in the spine, as they play a crucial role in trunk movement and supporting body weight. Structural deformations of the lumbar vertebrae, whether congenital or acquired, can lead to various diseases, the most common of which are adolescent idiopathic scoliosis and spondylolisthesis. Adolescent idiopathic scoliosis refers to the lateral curvature of the spine in adolescents aged 10 to 18 years and affects approximately 1 to 3% of adolescents in the USA and 0.5 to 5.2% worldwide [1, 2]. Spondylolisthesis occurs when one vertebra slips forward relative to the vertebra below, and its incidence is approximately 6% in the general population [3, 4]. X-ray, computed tomography, and magnetic resonance imaging (MRI) can be employed to diagnose these diseases. Among these modalities, X-rays are frequently used for the initial diagnosis because of their simplicity and cost-effectiveness [5]. Diagnosis has traditionally relied on the manual inspection of X-ray images by medical staff, including radiologists or orthopedists, to identify abnormalities. However, there has been a recent trend towards automating this process to reduce expenses and enhance hospital efficiency.

Several studies have been conducted to develop automatic analysis techniques for lumbar vertebrae or spinal X-ray images [6]. In recent years, deep-learning-based methods have emerged as dominant approaches, showing superior performance compared to traditional image-processing techniques. These deep-learning methods can distinguish abnormal X-ray images from normal images [7] and identify specific regions in an image for diagnostic purposes [8]. The latter approach offers the advantage of intuitive understanding and applying the same criteria as medical professionals in their judgments. More specifically, studies on this approach can be categorized into segmentation and landmark detection methods. Segmentation methods partition an image into regions of interest, which are primarily used to identify the shape of each vertebra. For example, a Mask R-CNN model was utilized to segment whole-spine vertebrae in lateral X-ray images [9]. Spinopelvic parameters were calculated from the segmented results. Peng et al. incorporated attention modules into a network based on U-Net [10]. The model discerned the contours of the vertebrae for scoliosis diagnosis. Chen et al. employed U-Net and Mask R-CNN for the segmentation of the cervical and lumbar vertebrae [11]. The two segmentation results were then merged using an ensemble rule. The introduced ensemble method outperformed other state-of-the-art methods in terms of segmentation accuracy. Compared to segmentation methods, landmark detection methods do not provide detailed information on vertebral shapes. However, these methods require relatively simple labeled data for model training. This can be a significant advantage in medical imaging, where label data acquisition is costly. The goal of landmark detection methods is to locate specific landmarks that represent the positions or morphological features of the vertebrae. Then, several parameters, including the Cobb angle and degree of slippage, are obtained to identify diseases [12, 13]. For instance, one study employed a cascaded pyramid network with differentiable spatial-to-numerical transformation layers in whole-spine lateral X-ray images to localize 45 anatomical landmarks and calculate 18 parameters to assess spinal balance [14]. The performance was validated using 400 images. The median localization errors ranged from 1.76 to 2.63 mm in the lumbosacral area, and the Pearson correlation coefficients between the calculated parameters and ground-truth values were higher than 0.9, except for one parameter. Another study focused on localizing the corners of the vertebrae to diagnose lumbar spondylolisthesis [15]. Two convolutional neural network (CNN) models were trained on 1000 images to detect and correct landmarks. The method was evaluated using the Meyerding classification, which represents the status of lumbar spondylolisthesis, and achieved 98% agreement with a doctor. Similarly, another study proposed LumbarNet, which combines U-Net and a feature fusion module [13]. The accuracy of vertebral slip detection was 88.83% based on the investigation of data from the Taipei Medical University Hospital. Another approach employed a two-step CNN architecture to detect the corners of the vertebrae [16]. The first CNN identifies each vertebra in an X-ray image, and the image is then cropped for each vertebra. Subsequently, the second CNN detects the four corner points and calculates the landmark coordinates. The dataset consisted of 10,193 instances, which were divided into training, validation, and testing sets. The median localization errors for the X- and Y-coordinates relative to the vertebral width were 1.98% and 1.68%, respectively. The application of deep-learning-based methods has significantly improved the performance of X-ray image analysis. However, errors can still occur in landmark detection, leading to misdiagnosis. The noise and ambiguity of images owing to overlap with other organs or bones can lead to errors, and the inherent variability in the shape of the lumbar vertebrae also makes it difficult to find landmarks [17].

Various techniques have been investigated to mitigate these errors and enhance the robustness of landmark detection in lumbar X-ray images. As one of them, landmark detection methods employing both local image features and the structural distribution properties of landmark points have been studied [18–20]. There may be a connection relation between two landmarks in a vertebra. The connection relationship information was utilized for a deep-learning model that assesses adolescent idiopathic scoliosis [18]. Non-directional part affinity fields (PAFs) containing connection relations were constructed and employed as additional target data in the training phase of the method. The effectiveness of this approach was demonstrated experimentally by comparing the landmark detection accuracy with that of other methods. However, the connection relation only encodes the pairing information between the left- and right-center landmarks, and this method does not fully utilize the distribution information of all landmarks. Another study addressed the issue of the erroneous detection of landmarks of the lumbar vertebrae and sacrum using the CoordConv operation [19]. This operation allowed the model to incorporate coordinate information during the detection process. It was confirmed that the introduced method contributed to a reduction in landmark detection errors. The geographical distribution of landmarks can provide valuable information for identifying landmarks, as demonstrated in a previous study [20]. Despite these benefits, this method has some limitations. It is difficult to understand how the model uses distribution information intuitively, and the influence of geographical information on the final results is not adjustable. Principal component analysis (PCA) was employed to analyze the statistical characteristics of landmark distributions. Subsequently, multistage cascaded CNNs were used to update the eigenvalues and landmark locations iteratively. This method exhibited improvements in error reduction. However, it used the distribution characteristics of only four landmarks within the same vertebra for the final position correction, instead of the entire landmark distribution. This method repeatedly performs the overall process, including image analysis and landmark updating, thereby increasing its complexity. In addition, the three aforementioned techniques require unique network structures or learning methodologies that are tailored specifically for additional target data. For example, additional target data representing the relationship between two landmarks should be constructed to use PAFs, and a network must contain CoordConv layers that process the coordinate information of a target for the CoordConv operation [19]. The PCA-based method provides eigenvalues relating to the distribution of landmarks [20]. Therefore, the specific structure of the network must be trained to generate eigenvalue results. While these features are not problematic when a method is used independently, they may become limitations when combined with other landmark correction techniques or when a current backbone model is replaced with a more effective one.

In this study, a heatmap-based active shape model (HASM) method is proposed to improve the robustness of landmark detection in an X-ray image by leveraging the distribution characteristics of all landmark points without constraints on the model architecture. The HASM investigates the heatmap results of a CNN-based model [19] and corrects the initial landmark positions using the active shape model (ASM). A heatmap is an image result that presents the local response of a target and is generally used as the output result of a CNN-based model that performs localization tasks. Therefore, the heatmap result is commonly obtained from deep-learning models for analyzing lumbar X-ray images, and landmarks are detected as points with the local maximum response in the heatmap [14–16, 18, 19]. The ASM is used to revise the initial landmark positions using a shape model that contains the statistical characteristics of the landmark distribution. In the correction process, the ASM updates the position of each landmark by applying a certain criterion and rearranges all landmarks to ensure that they do not violate the shape model [21]. Typically, the edge or two-dimensional (2D) patch image information is employed as a criterion to update the position of each landmark in the ASM. ASMs have been implemented in various applications including medical image processing [22, 23]. Several recent studies have attempted to integrate ASMs with deep-learning models to leverage the strengths of both techniques [24–26]. Hsu utilized Faster R-CNN to segment the left ventricle in cardiac ultrasound image sequences [24]. The initial segmentation results were refined using the ASM. He et al. introduced a CNN-based method for identifying the prostate in MRI [25]. In this method, the result of a CNN model is considered as a boundary probability map and the ASM is applied to update the current contour to the maximum probability points. For detection tasks rather than segmentation, one study used the outputs of a CNN model as a feature map for the ASM in facial landmark detection [26]. This demonstrated the effectiveness of the CNN-based feature map compared with conventional features such as the histogram of oriented gradients, scale-invariant feature transform, and texture templates. Similar to previous research, the proposed HASM assumes that heatmap results are generated from a CNN-based model and applies an ASM based on the heatmap to leverage the image analysis capabilities of the deep-learning model and incorporate the statistical information of landmark distribution. To the best of our knowledge, this study is the first to employ a deep-learning and ASM-based approach to analyze lumbar vertebrae in X-ray images. This method has several distinctive characteristics tailored to the purpose of the analysis, including data augmentation for constructing the shape model and landmark modification using multiple heatmap results. These features are described and compared in detail in Sections “Materials and Methods” and “Discussion”.

## Materials and Methods

### Deep-Learning Model to Generate Heatmap Results

**Fig. 1:**
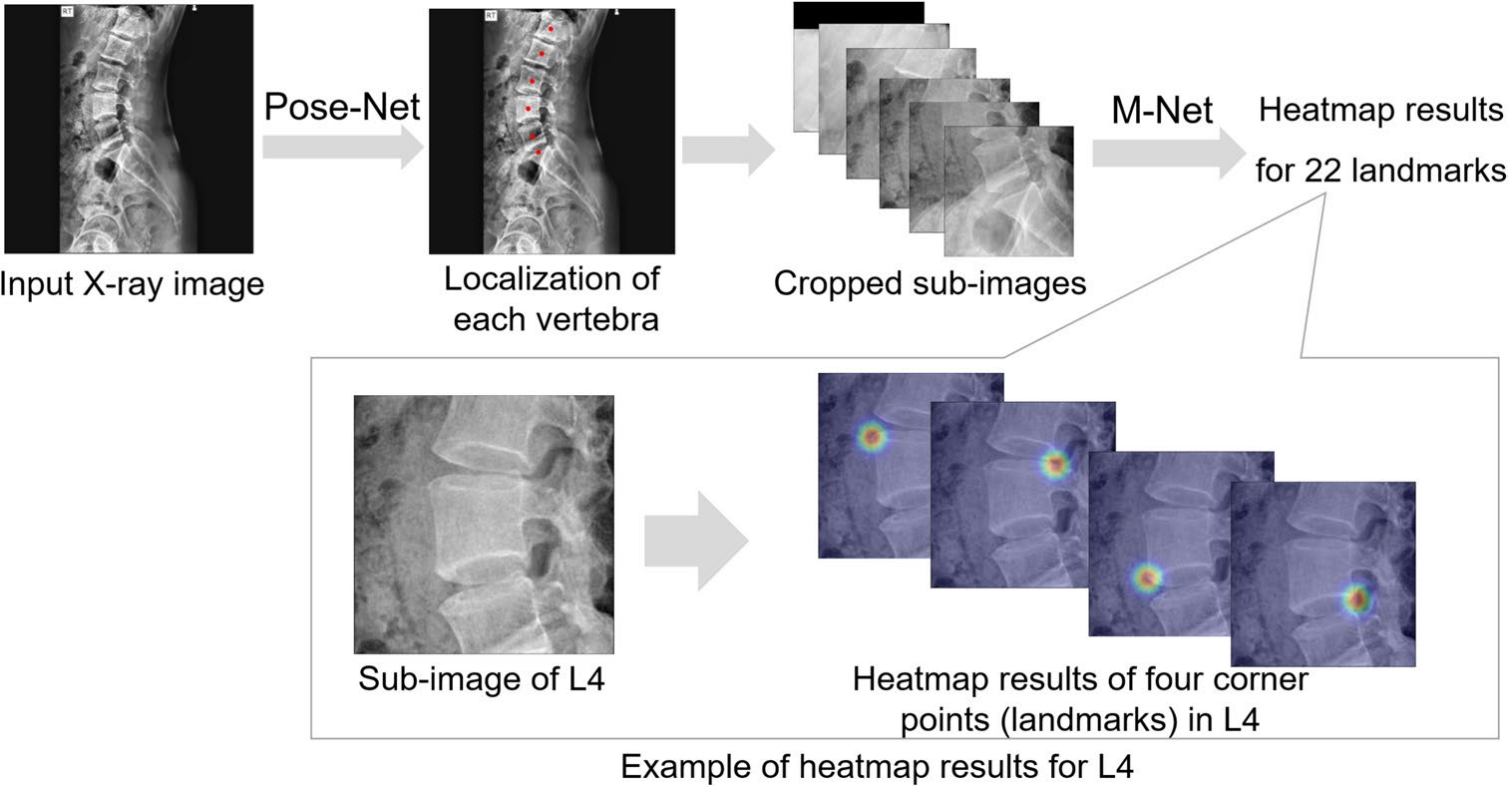
Two-step deep-learning model to generate heatmap results containing information on landmark positions

**Fig. 2:**
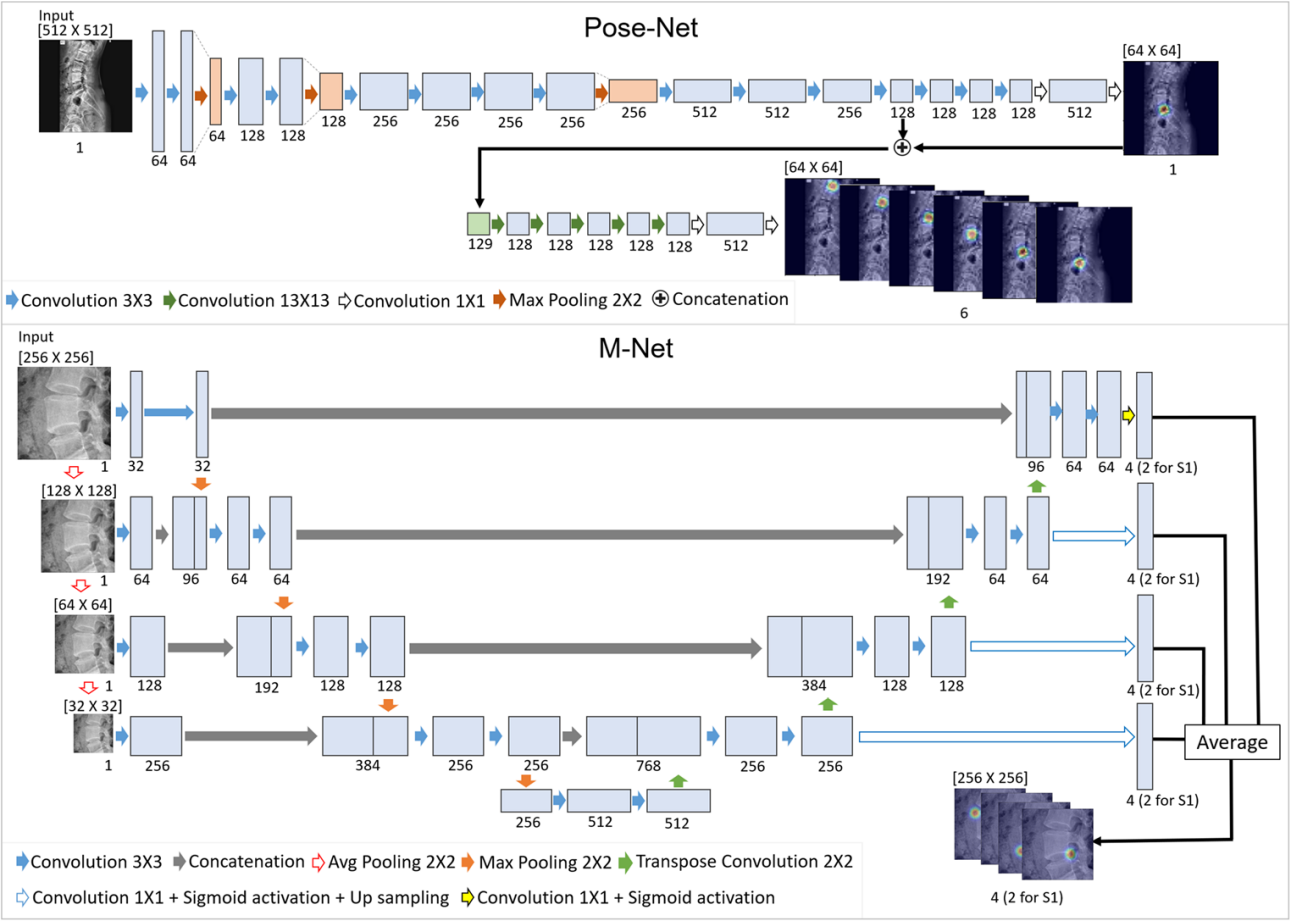
Structure of the deep-learning model consisting of Pose-Net and M-Net

In this study, landmarks are defined as the four corner points of each of the five lumbar vertebrae (L1 to L5) and the two endpoints on the upper line of the sacrum (S1). Initially, 22 landmarks are detected in a lateral X-ray image using a CNN-based deep-learning model, and the landmark positions are corrected using the proposed HASM. The deep-learning model utilized in this study employs a two-step approach involving Pose-Net and M-Net (Fig. 1). Pose-Net analyzes the X-ray image as a whole and localizes the center points of L1 to L5 and the upper endplate line of S1. The structure is shown in the upper part of Fig. 2. The input image is padded and resized to $512 \times 512$, and consecutive convolutional blocks are used for the image analysis. Each convolutional block has instance normalization, ReLU activation, and a convolutional layer. The size of the receptive field and number of filters for the convolutional layer are specified as text labels in the figure. Among L1 to L5, L5 is the most easily distinguishable because of its proximity to S1. Therefore, Pose-Net prioritizes the determination of the location of L5 and estimates the locations of the other vertebrae based on this information. The output of Pose-Net is a six-channel heatmap response corresponding to L1 to L5 and S1. The original image is then divided into six sub-images according to the Pose-Net result. M-Net examines these sub-images and generates heatmap results for the 22 landmarks. M-Net constructs multiscale images from an input and employs multiple levels of receptive fields of various sizes. The analyzed multi-scale results are merged using an average operation. These characteristics allow M-Net to better capture the local and global contextual information of landmarks, which is advantageous for investigating vertebrae of diverse shapes and scales, as seen in previous comparative studies on lumbar X-ray images [27, 28]. In this study, the convolutional block of M-Net is equivalent to that of Pose-Net in terms of its structure. In X-ray image analysis, the consideration of noise, object positioning, and anatomical characteristics is crucial. Previous studies have employed data augmentation techniques to train a model by introducing diverse variations into the input images [19, 27]. To improve the robustness of image interpretation, this approach is also adopted in this study, and the input images are transformed using random spine cutout, brightness, contrast, scaling, and rotation of up to 30 degrees to train the models. In addition, Gaussian distribution heatmaps corresponding to the true locations of the landmarks are generated and utilized during the model training process. The remaining details are the same as those in the referenced research [19], but the use of PAFs and the CoordConv operation are excluded to verify the effectiveness of the HASM by removing other factors that could influence landmark detection performance. However, the simulation results of the HASM for the model containing PAFs and the CoordConv operation are also presented in Section “Comparison and Integration with Other Methods” to demonstrate the effect of the HASM under various conditions.

**Fig. 3:**
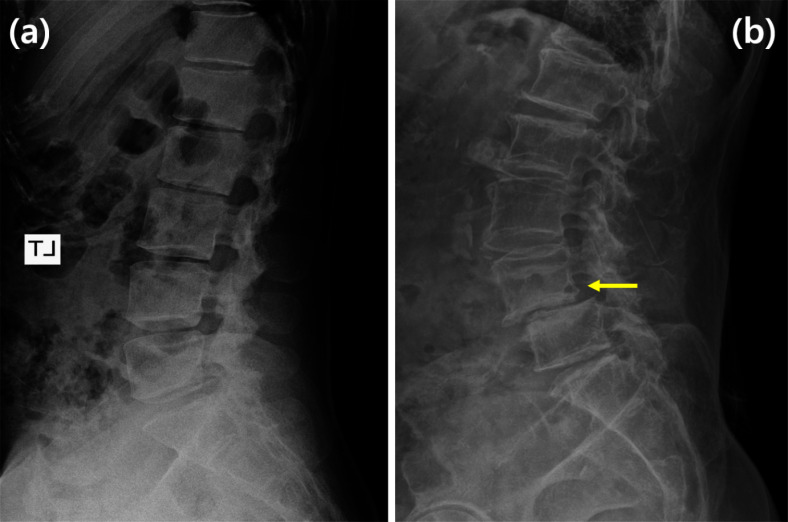
Examples of X-ray images: **a** normal condition and **b** spondylolisthesis

**Fig. 4:**
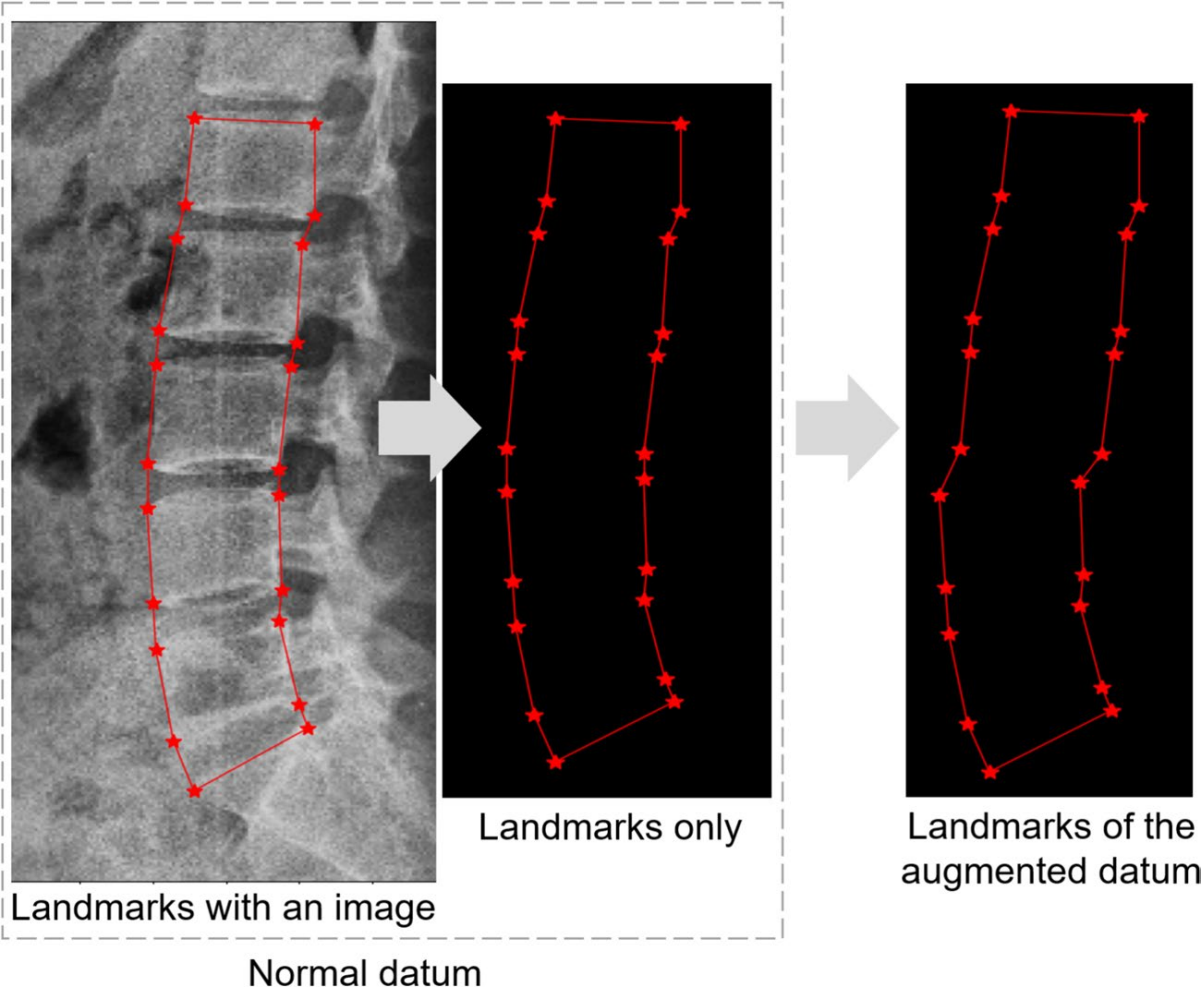
An example of artificial data augmentation ($V_{start}$ = 4, $V_{end}$ = 6, $\alpha$ = -1, $\beta$ = 0.15). The red stars are landmark positions

### Construction of Shape Model

The HASM utilizes the statistical properties of the landmark distribution to revise landmark positions. The shape model, which represents the statistical information of the landmarks, was extracted by analyzing the training data of the deep-learning model. Let the coordinates of the *i*-th landmark be denoted as ($x_i$, $y_i$) and the positions of the 22 landmarks be expressed as a vector with 44 elements ($x_1$, $x_2$,..., $x_{22}$, $y_1$,..., $y_{22}$). The vectors from the entire training dataset are aligned in the same coordinate system using generalized Procrustes analysis, and PCA is performed on the aligned vectors [29]. The resulting shape model includes the mean vector and eigenvectors obtained from PCA.

The shape model represents the data characteristics; however, it may be biased towards normal data from individuals without any diseases such as spondylolisthesis. Figure 3 shows an example of spondylolisthesis; the misalignment between L4 and L5 is indicated by the yellow arrow in (b). This bias arises owing to an imbalance in the amount of normal and spondylolisthesis data and increases as the amount of normal data increases compared to the spondylolisthesis data. Such data imbalance is common in real-world scenarios, and the landmarks of spondylolisthesis data may be incorrectly adjusted using a biased shape model, as if they were normal data. To address this issue, the proposed method artificially augments the spondylolisthesis data based on the data imbalance. Algorithm 1 describes the augmentation process, which generates artificial spondylolisthesis data from normal data. The augmentation process involves the parallel movement of a series of consecutive vertebrae to create a landmark distribution similar to that in spondylolisthesis. This is achieved using four parameters: $V_{start}$, $V_{end}$, $\alpha$, $\beta$. When assigning numbers 1 to 6 to vertebrae L1 to L5 and S1, $V_{start}$ and $V_{end}$ are used to select the consecutive vertebrae to be moved. Subsequently, the vertebrae are moved forward or backward according to $\alpha$. The movement distance is determined by $\beta$ and the distance between the top two points of the first vertebra to be moved. The range of $\beta$ is from 0 to $\beta _{max}$, and 0.3 is used for $\beta _{max}$ in this method. Figure 4 shows an example of the augmentation process, where $V_{start}$ and $V_{end}$ are set to 4 and 6, respectively, resulting in the movement of landmarks from L4 to S1 to generate an artificial spondylolisthesis case. This augmentation process enhances the diversity in the data distribution and enables the construction of a shape model that is suitable for both normal and spondylolisthesis data. This process is not for generating images, but for generating coordinates of landmarks in the same form as the labels of the training data that represent landmark positions. A landmark distribution similar to that of spondylolisthesis is created through the simple manipulation. The augmented data do not fully represent the medical characteristics of spondylolisthesis; however, they reflect information on more diverse landmark distribution cases in the shape model. It should be noted that the method assumes the availability of X-ray images with spondylolisthesis information in the training data. However, when the training data do not include spondylolisthesis labels, a certain proportion of the data can be selected and processed for augmentation.

**Algorithm 1:**
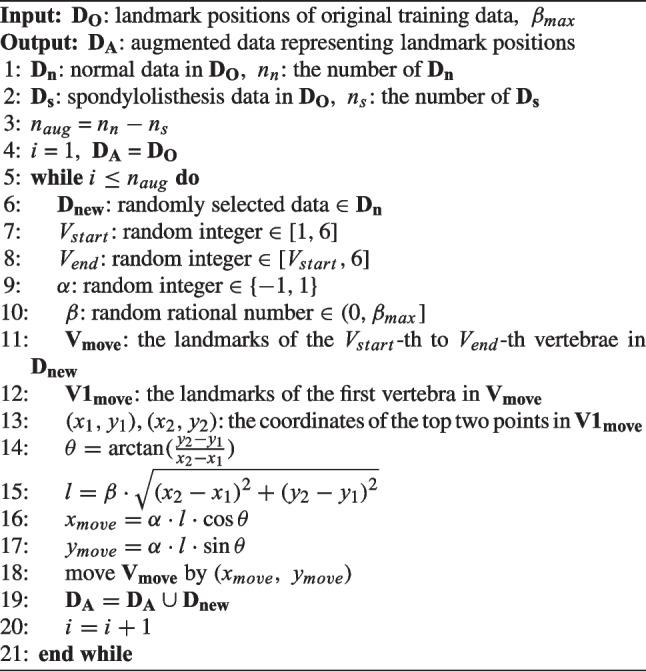
Augmentation of spondylolisthesis data

### Heatmap-Based Active Shape Model

Landmarks are initially detected as the points with the highest response in each heatmap generated by the deep-learning model. Subsequently, the proposed HASM corrects the identified landmarks through a two-step iterative process: updating the position of each landmark and reorganizing the entire set of landmarks based on the shape model. The iteration process is performed a predetermined number of times or terminated early when the changes in landmark positions are not significant. In this study, the number of iterations was 30; related information can be found in the Supplementary Material 1.

**Fig. 5:**
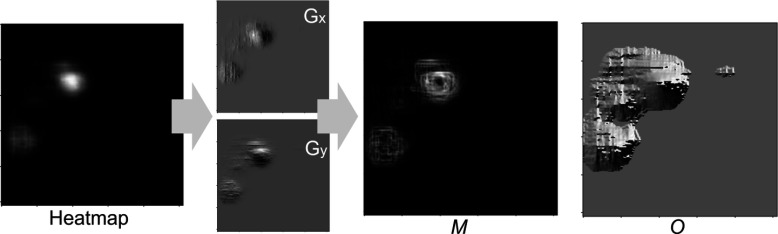
Calculation of gradient information from the heatmap response of a landmark

The conventional ASM updates the position of each landmark using image analysis techniques based on the edge or 2D patch image information [29]. However, the use of traditional techniques may diminish the effectiveness of the deep-learning model. This may have adverse effects when correcting landmarks because deep-learning models generally outperform traditional image analysis techniques. To address this limitation, the proposed HASM utilizes the heatmap results from the deep-learning model to update each landmark. This approach maximizes the utilization of the exceptional image analysis capabilities of the deep-learning model. Algorithm 2 provides a detailed description of the process. The heatmap response can be interpreted as the probability or likelihood of the landmark position. This process examines the gradient of the heatmap response at the current landmark positions and adjusts the landmarks, moving them towards regions with higher response values based on the gradient information. The gradient information indicates that the direction and magnitude of the heatmap response increase at each point. This information is used to update the current landmark location by reflecting the heatmap response, but not to analyze the morphological characteristics of the heatmap itself, such as finding edges. To calculate the gradient information of the heatmap response, a Sobel filter is employed, and the magnitude (${\textbf {M}}$) and orientation (${\textbf {{O}}}$) of the heatmap gradients are obtained (Fig. 5). Let $m_h$ and $o_h$ be the values at the current positions of the landmarks in the ${\textbf {M}}$ and ${\textbf {O}}$, respectively. These values determine the distance and direction for updating the landmark position from the current position (${\textbf {CL}}$). The sensitivity of the movement, which is based on the heatmap gradient, is controlled by a user parameter $\mu$, and the procedure for setting the parameter is described later.

**Algorithm 2:**
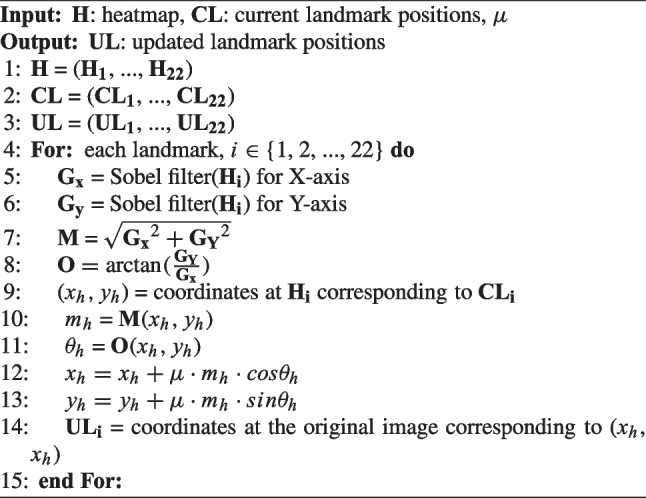
Updating the position of each landmark

The updated landmark positions (${\textbf {UL}}$) are then rearranged using the shape model, following the same process as in the conventional ASM. Algorithm 3 provides a brief overview of the procedure; more detailed information can be found in the literature [21]. Let $\bar{{\textbf {x}}}$ and ${\textbf {P}}$ represent the mean vector and eigenvectors for the first $n_P$ eigenvalues in the shape model, respectively. ${\textbf {UL}}$ is reshaped into a vector ${\textbf {Y}}$. Subsequently, the re-arrangement process verifies and adjusts ${\textbf {Y}}$ to ensure that it maintains the statistical characteristics of the landmark distribution represented by $\bar{{\textbf {x}}}$ and ${\textbf {P}}$. This process results in a vector X, minimizing the sum of the square distances to ${\textbf {Y}}$ while preserving the characteristics of the shape model. ${\textbf {X}}$ is constructed by finding a shape vector ${\textbf {b}}$. Initially, ${\textbf {b}}$ is set to a zero vector, which is used to determine a vector ${\textbf {x}}$, as described in line 4 of Algorithm 3. ${\textbf {Y}}$ is then transformed into the coordinate frame of ${\textbf {x}}$ and projected into the tangent plane to $\bar{{\textbf {x}}}$, resulting in ${\textbf {y'}}$. ${\textbf {b}}$ can be re-obtained as described in line 8 using ${\textbf {y'}}$. ${\textbf {b}}$ is updated gradually through iterative operations, and the final ${\textbf {x}}$ is calculated from the converged ${\textbf {b}}$. Finally, ${\textbf {x}}$ is transformed back into the image coordinate frame as ${\textbf {X}}$. The rearranged ${\textbf {UL}}$ is obtained from ${\textbf {X}}$ and is again assigned as ${\textbf {CL}}$.

**Algorithm 3:**
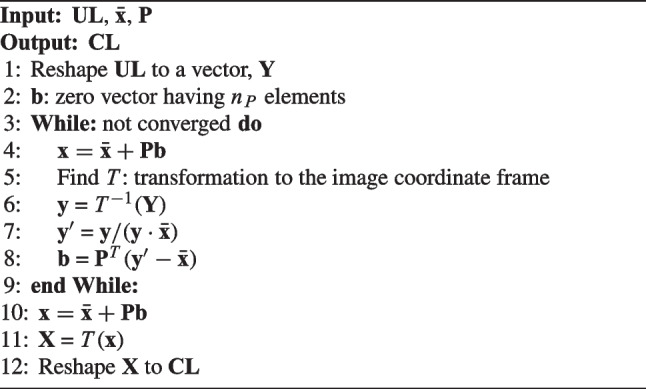
Reorganizing the set of landmarks based on the shape model

**Fig. 6:**
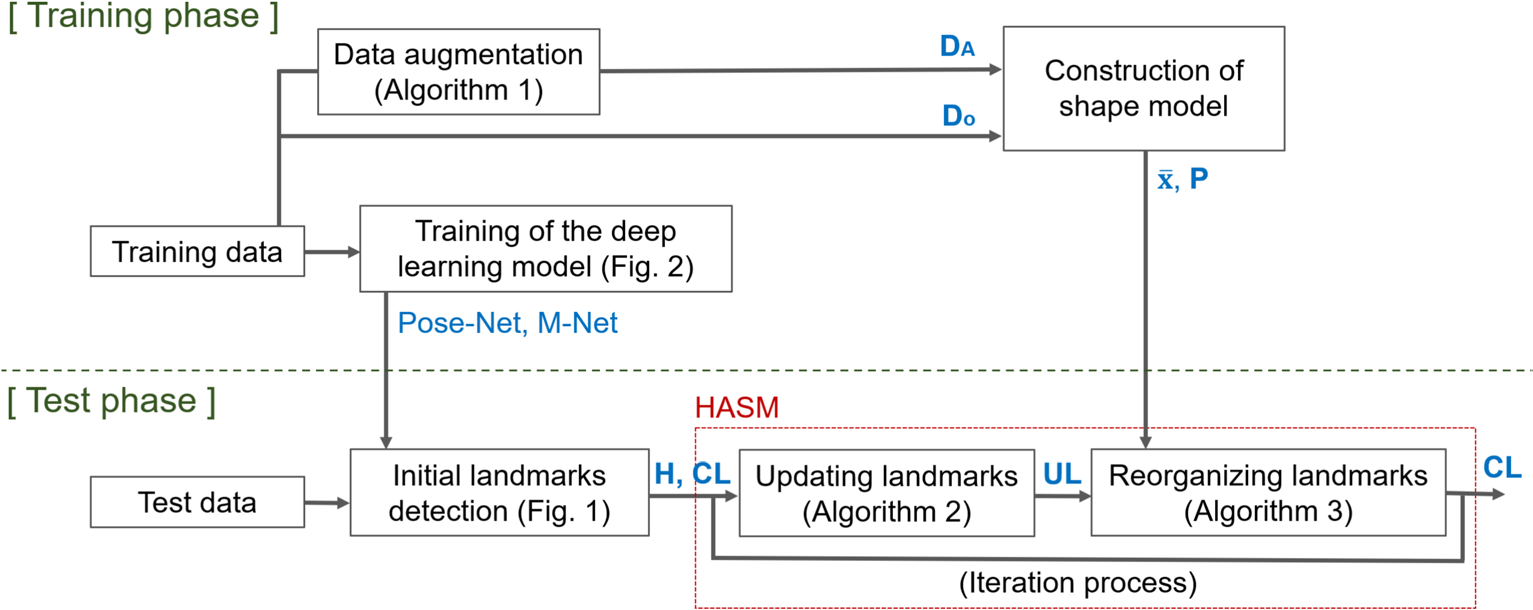
Overall data flow diagram of the proposed method

Initially, the landmarks are identified as the points with the highest heatmap responses, and the amount of updates is insignificant. However, because the landmarks are rearranged using a shape model, which contains the statistical attributes of the landmark distribution, the landmark positions deviate from the points with the global maximum response. After the correction process is performed using the shape model, the heatmap-based update repositions the landmarks in the direction of increasing the heatmap response, reflecting the analysis results of the deep-learning model. Through the iterative application of these two processes, the positions of the landmarks are adjusted to incorporate both the image analysis capability of the deep-learning model and the statistical characteristics of the landmark distribution. Figure 6 shows the data flow diagram and represents the role of the HASM in the overall process of detecting landmarks. As mentioned previously, the shape model and deep-learning model are constructed using training data. The landmarks of the test data are initially detected by the deep-learning model. The proposed HASM, as described in Algorithms 2 and 3, refines these results to determine the final landmark positions.

## Results

### Dataset

The BUU spine dataset, constructed by Burapha University, was used for the evaluations [30]. This dataset contains X-ray images of anteroposterior and lateral views of 3600 subjects. This study proposes a landmark detection method for lateral X-ray images. The results can be utilized to analyze spondylolisthesis, which is our future research topic. Therefore, all 3600 images of the lateral lumbar spine were used in the simulations (Table 1). All images contained vertebrae from L1 to S1, and the proposed method assumes that it is applied to images in which all landmarks from L1 to S1 are present. The age of the subjects ranged from 6 to 97 years, and 1320 of them were male. Two important parameters affect the quality of X-ray images. One is the X-ray tube voltage, and the other is the X-ray tube current and exposure time; values of 80 to 90 kV and 50 to 60 mAs were used for these parameters, respectively. The dataset included three labels: the position of the lumbar vertebrae, spondylolisthesis diagnosis, and presence of lumbosacral transitional vertebrae. The positions of the lumbar vertebrae consisted of 22 points, as described in Section “Deep-Learning Model to Generate Heatmap Results”. The positional information was used as the ground truth for this study. Our previous study provides the detailed criteria for defining the positions of these landmarks [31]. The goal of this study was to detect landmarks in X-ray images. Therefore, this study did not utilize other diagnostic information, except for spondylolisthesis information for the data augmentation process to generate the shape model. However, spondylolisthesis information is not essential for implementing the proposed method, as mentioned in Section “Construction of Shape Model”. To construct label information, the collected images were reviewed and aligned in the same direction by computer vision experts. Radiologists and orthopedists then inspected the images to generate label information. The final labels were finalized through a meeting among computer vision experts, radiologists, and orthopedists. More detailed information on the data construction process and example figures can be found in the related research paper [32].

**Table 1:** Information of utilized image: range, mean, and standard deviation (SD)

Image characteristics	Range	Mean	SD
Height (pixel)	1752–3408	2490.696	220.816
Width (pixel)	868–3040	1812.633	382.966
Pixel spacing (mm)	0.125$-$0.175	0.170	0.012

### Simulation Environment and Performance Indices

The effectiveness of the proposed HASM was evaluated using a computer equipped with an Intel i9-12900KS CPU, 128 GB of RAM, and an NVIDIA GeForce RTX 3090 Ti GPU. The simulations and implementations of the deep-learning model and HASM were conducted using Python and PyTorch. Hold-out validation was employed to assess the landmark detection performance. The dataset of 3600 images with label information, as described in Section “Dataset”, was randomly divided into training, validation, and test sets comprising 70%, 10%, and 20% of the data, resulting in 2520, 360, and 720 images in each set, respectively. The designated single test set was consistently investigated across all simulations. The training data were used to construct the deep-learning model and shape model for the HASM. The validation data were utilized to monitor the training process of the deep-learning model and to determine two parameters of the HASM: $n_P$ and $\mu$. The hyperparameters of the deep-learning model were designated based on the results of a previous study [19]. The learning rates were 0.001 and 0.0001, and the batch sizes were 16 and 32 for Pose-Net and M-Net, respectively. Training was conducted for 300 epochs using the Adam optimizer. The learning rate was decreased linearly according to the number of epochs, and the training process was terminated early if there was no improvement in the validation loss for 25 consecutive epochs. The parameter $n_P$ represents the number of eigenvectors used to describe the potential distribution of landmarks and imposes more constraints on the distribution as the value decreases from 44. The parameter $\mu$ determines the sensitivity of movement in the landmark position update. The amount of position change increases with a larger $\mu$ value. A grid was composed for the two parameters, and a pair of parameters was selected for the HASM using a grid search method based on the validation data in each simulation [33]. The designated values for $n_P$ and $\mu$ were 20 and 0.02, respectively, in the proposed HASM. More detailed simulation results for the selection of the two parameters are presented in the Supplementary Material 2.

To evaluate the changes after applying the proposed HASM, the averaged maximum errors ($AE _{max}$) and averaged mean errors ($AE _{mean}$) were calculated as follows:

1 $$\begin{aligned} E_{ij} = \sqrt{ (\hat{x}_{ij} - x_{ij} )^2 + (\hat{y}_{ij} - y_{ij})^2 }, \end{aligned}$$

2 $$\begin{aligned} E_{max,i} = \max _j (E_{ij}), \end{aligned}$$

3 $$\begin{aligned} E_{mean,i} = \frac{1}{22} \sum _{j=1}^{22} E_{ij}, \end{aligned}$$

4 $$\begin{aligned} AE _{max} = \frac{1}{N} \sum _{i=1}^{N} E_{max,i}, \end{aligned}$$

5 $$\begin{aligned} AE _{mean} = \frac{1}{N} \sum _{i=1}^{N} E_{mean,i}, \end{aligned}$$

where ($x_{ij}, y_{ij}$) and ($\hat{x}_{ij}, \hat{y}_{ij}$) represent the detected and ground-truth positions, respectively, of the *j*-th landmark in the *i*-th image out of the total *N* images. The error is first calculated as the pixel distance, which quantifies the differences between the detected and ground-truth landmark positions in the coordinate frame of the X-ray images. The value is then converted into millimeters to represent the actual error values in a real coordinate frame. In landmark detection studies, $AE _{mean}$ is generally used to verify a method [14]; however, $AE _{max}$ was also used in this study. This is because the proposed method focuses on correcting initially detected landmarks, and its effectiveness is substantial only for certain data with large initial errors, rather than reducing the errors overall. Therefore, the improved robustness of landmark detection using the proposed method was demonstrated using this index, which is consistent with other studies with similar purposes [34, 35]. In addition, the allowable error ($E_{a,i}$) was computed to compare the error relative to the size of the vertebrae in each image. $E_{a,i}$ was defined as 20% of the average length of the vertebral edges and is calculated by the following:

6 $$\begin{aligned} l_{j1} = \sqrt{ (x_{UL,j} - x_{UR,j})^2 + (y_{UL,j} - y_{UR,j})^2}, \end{aligned}$$

7 $$\begin{aligned} l_{j2} = \sqrt{ (x_{UL,j} - x_{LL,j})^2 + (y_{UL,j} - y_{LL,j})^2}, \end{aligned}$$

8 $$\begin{aligned} l_{j3} = \sqrt{ (x_{UR,j} - x_{LR,j})^2 + (y_{UR,j} - y_{LR,j})^2}, \end{aligned}$$

9 $$\begin{aligned} l_{j4} = \sqrt{ (x_{LL,j} - x_{LR,j})^2 + (y_{LL,j} - y_{LR,j})^2 }, \end{aligned}$$

10 $$\begin{aligned} E_{a,i} = \frac{1}{5} [\frac{1}{21}\{\sum _{j=1}^{5}(l_{j1}+l_{j2}+l_{j3}+l_{j4}) + l_{61}\} ], \end{aligned}$$

where $(x_{UL,j}, y_{UL,j}), (x_{UR,j}, y_{UR,j}), (x_{LL,j}, y_{LL,j})$, and $(x_{LR,j}, y_{LR,j})$ denote the coordinates of the upper-left, upper-right, lower-left, and lower-right points of the *j*-th vertebra, respectively. Subsequently, the normalized $AE _{max}$ and $AE _{mean}$ ($NE _{max}$ and $NE _{mean}$) were computed using the following equations:

11 $$\begin{aligned} NE _{max} = \frac{1}{N} \sum _{i=1}^{N} \frac{E_{max,i}}{E_{a,i}}, \end{aligned}$$

12 $$\begin{aligned} NE _{mean} = \frac{1}{N} \sum _{i=1}^{N} \frac{E_{mean,i}}{E_{a,i}}, \end{aligned}$$

and $E_{a,i}$ was also utilized to investigate the error distribution in each simulation. The utilized dataset had various image sizes, and the proportion of lumbar vertebrae in each image varied owing to individual physical characteristics. These normalized indices allow for the comparative analysis of results by eliminating image-dependent factors [19, 20, 35].

### Evaluation of HASM

Tables 2 and 3 present the results of simulations conducted to evaluate the benefits of the proposed HASM. The landmarks of 720 X-ray images were detected using the deep learning model, and performance indices for the detection error were calculated for two cases: when the proposed HASM was not used ($wo\_HASM$) and when it was used ($w\_HASM$). The case without the data augmentation technique ($w\_HASM_{woDA}$) was also simulated to verify the effect of the shape model constructed by augmenting spondylolisthesis data. The $AE _{max}$, $AE _{mean}$, $NE _{max}$, and $NE _{mean}$ were obtained for each method. In addition, intervals were defined using integer multiples of $E_{a,i}$, and the data distribution was investigated based on the $E_{max,i}$ value of each data for comparison purposes. The results demonstrate that the proposed HASM was effective in enhancing the robustness of landmark detection. The HASM successfully corrected the detection errors of the deep-learning model, and the amount of data with an $E_{max,i}$ value larger than $E_{a,i}$ was reduced (Table 2). Error reduction occurred not only at specific intervals but also in all regions, including intervals for large errors exceeding $3E_{a,i}$. This demonstrates the effectiveness of the proposed HASM for addressing cases with large errors. The number of data in the interval [$\le E_{a,i}$] increased in $w\_HASM$ owing to the decreased errors. There were 29 more data in this interval compared to those of $wo\_HASM$, accounting for 4.03% of the entire test dataset. In the previous study that determined the ground truth of landmark positions using the same standard as the BUU spine dataset used in this study [31], the labeling deviation of 12 medical professionals ranged from 1.185 to 1.670 mm for the landmarks in L1 to L5 on average. This error range indicates the degree of error that can occur even when a medical professional performs the examination directly. The average value of $E_{a,i}$ for the test data was 7.0529 mm in this study. The value of $E_{a,i}$ can be considered a significantly large error for an automatic image analysis method aimed at replacing medical professionals, even considering the large amount and diversity of data used as well as the inclusion of the analysis results for S1 in this study. Therefore, it is important to reduce large errors more than $E_{a,i}$ in terms of the image analysis stability, and as shown in Table 2, the HASM demonstrated its advantage in this regard. Furthermore, $AE _{max}$ and $NE _{max}$ decreased by 5.58% and 5.65%, respectively, in $w\_HASM$ compared to $wo\_HASM$ (Table 3). The absolute magnitude of the error reduction was not substantial because the HASM primarily refined the landmark detection results obtained from the deep-learning model. However, considering that the amount of data in the interval [$\le E_{a,i}$] was changed by only 4.03% of the entire data, the influence of the HASM was meaningful for the data that required correction. The reductions in $AE _{max}$ and $NE _{max}$ showed statistical significance with *p* < 0.001. These findings also demonstrate that the HASM can improve landmark detection results using a deep-learning model. In contrast, $AE _{mean}$ and $NE _{mean}$ increased after applying the HASM. The HASM utilizes a shape model together with heatmap information to correct landmark positions, and subtle positional variations in the overall landmarks occur when employing the HASM. This causes the positions of the landmarks to deviate slightly from the maximum response points of the heatmap, even for landmarks that do not require positional correction. The slight increases in $AE _{mean}$ and $NE _{mean}$ were caused by this phenomenon. However, the increase in $AE _{mean}$ was less than 0.05 mm, and the increased $AE _{mean}$ and $NE _{mean}$ were not huge compared to the changes in $AE _{max}$ and $NE _{max}$; the increased values accounted for only 6.92% and 6.63% of the decreased $AE _{max}$ and $NE _{max}$, respectively, after applying the HASM. In addition, the use of a post-processing method to adjust this phenomenon is discussed in Section “Use of Post-Processing”. The usefulness of the data augmentation method for the shape model was verified by comparing the error indices of $w\_HASM_{woDA}$ and $w\_HASM$. The amount of data in [$\le 2E_{a,i}$] was larger in $w\_HASM_{woDA}$, but a large error case in [$>5E_{a,i}$] could be corrected to smaller one in $w\_HASM$. In addition, other indices, including $AE _{max}$, $AE _{mean}$, $NE _{max}$, and $NE _{mean}$, were better, and the statistical significance of $AE _{max}$ and $NE _{max}$ was stronger in $w\_HASM$ than in $w\_HASM_{woDA}$. The augmentation of spondylolisthesis data provided additional information regarding the possible distribution of landmarks to the shape model, and the landmarks were corrected more stably because of this advantage.

**Table 2:** Analysis of data distribution based on the value of $E_{max,i}$ for each method

Method	$\le E_{a,i}$	$\le 2E_{a,i}$	$\le 3E_{a,i}$	$\le 4E_{a,i}$	$\le 5E_{a,i}$	$>5E_{a,i}$
$wo\_HASM$	586	76	15	4	2	37
$w\_HASM_{woDA}$	615	62	7	0	0	36
$w\_HASM$	615	63	7	0	0	35

**Table 3:** Changes in the performance indices of the landmark detection after applying the proposed HASM

Method	$AE _{max}$ (mm)	$AE _{mean}$ (mm)	$NE _{max}$	$NE _{mean}$
$wo\_HASM$	11.2491	3.1287	1.6026	0.4492
$w\_HASM_{woDA}$	10.8024$^{*}$	3.1894$^{*}$	1.5407	0.4579$^{*}$
$$w\_HASM$$	10.6209$$^{**}$$	3.1722$$^{**}$$	1.5121$$^{**}$$	0.4552$$^{**}$$

*p < 0.05 and **p < 0.01 in paired samples t-test with $wo\_HASM$

The BUU spine dataset includes labeled information on spondylolisthesis. Using this information, the effectiveness of the HASM for normal and spondylolisthesis data was compared, as shown in Tables 4 and 5; the number of data was 609 and 111 for normal and spondylolisthesis data, respectively. The overall trend was similar to that shown in Tables 2 and 3. By applying the HASM, the number of cases with errors larger than $E_{a,i}$ was reduced in both the normal and spondylolisthesis data. The $AE _{max}$ and $NE _{max}$ also improved. As the training data for the deep-learning model had a higher proportion of normal data, the errors in the spondylolisthesis data were higher than normal. The HASM also uses heatmap information, which is the result of the deep-learning model; therefore, this phenomenon did not change after applying the HASM. The number of data in the interval [$\le E_{a,i}$] was increased after applying the HASM, and the ratio was 4.11% and 3.60% for the normal and spondylolisthesis data, respectively. The value was lower in the spondylolisthesis data, but the difference was not meaningful. In contrast, the ratios of decreased $AE _{max}$ were 4.92% and 8.63% for the normal and spondylolisthesis data, respectively. The spondylolisthesis data achieved a more substantial error reduction rate despite exhibiting a more limited change in the interval [$\le E_{a,i}$] compared to the normal data. These results indicate that the $E_{max,i}$ reduction effect of the HASM was larger for the spondylolisthesis data on average. This was because the spondylolisthesis data were more likely to have larger errors, making the corrections introduced by the HASM more noticeable.

**Table 4:** Error distribution with respect to normal and spondylolisthesis data

Data	Method	$\le E_{a,i}$	$\le 2E_{a,i}$	$\le 3E_{a,i}$	$\le 4E_{a,i}$	$\le 5E_{a,i}$	$>5E_{a,i}$
Normal	$wo\_HASM$	503	59	12	4	1	30
	$w\_HASM$	528	45	7	0	0	29
Spondylolisthesis	$wo\_HASM$	83	17	3	0	1	7
	$w\_HASM$	87	18	0	0	0	6

**Table 5:** Changes in the performance indices for normal and spondylolisthesis data

Data	Method	$AE _{max}$ (mm)	$AE _{mean}$ (mm)	$NE _{max}$	$NE _{mean}$
Normal	$wo\_HASM$	10.9126	3.0453	1.5530	0.4365
	$$w\_HASM$$	10.3760$$^{**}$$	3.0867$$^{**}$$	1.4761$$^{**}$$	0.4422$$^{**}$$
Spondylolisthesis	$wo\_HASM$	13.0956	3.5868	1.8746	0.5186
	$w\_HASM$	11.9651$^{*}$	3.6416	1.7098$^{*}$	0.5266

*p < 0.05 and **p < 0.01 in paired samples t-test with $wo\_HASM$ of the same data (normal or spondylolisthesis data)

**Fig. 7:**
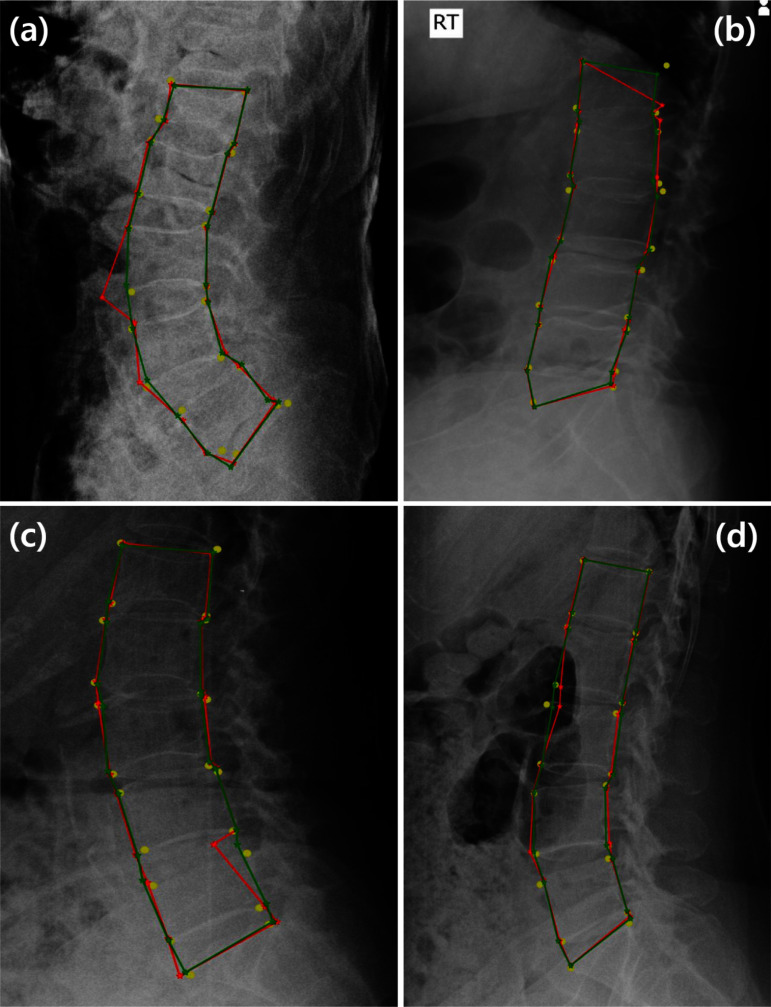
Examples of landmark detection errors and their corrections: **a** a large error in the lower-left point of L3, **b** an undetected upper-right point in L1, **c** an image containing noise components, and **d** unclear vertebrae owing to interference from other organs. The yellow circles represent the ground-truth positions of the landmarks. The red stars are landmarks detected by the deep-learning model, and the green stars are corrected landmarks after applying the proposed HASM. The red and green lines connect the detected landmarks for visualization

**Fig. 8:**
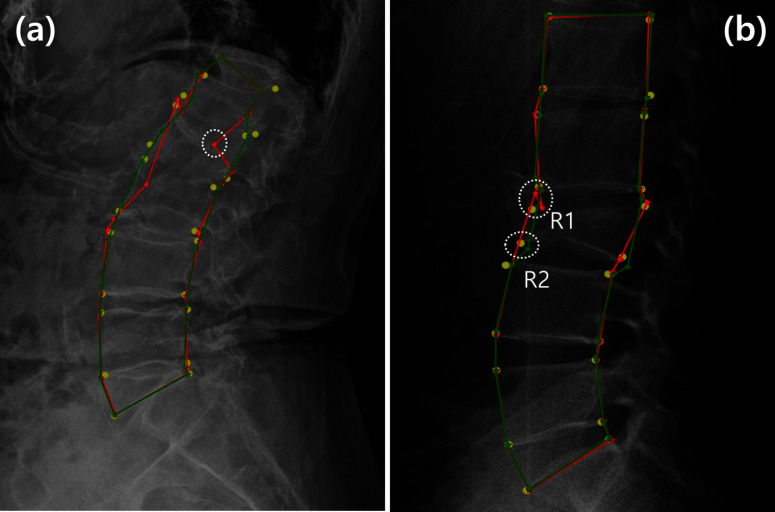
Cases in which the proposed method could not achieve effective improvement: **a** unclear vertebrae owing to the influence of body tissues and **b** a morphological abnormality of L3

### Example Cases Representing Effect of HASM

Figure 7 shows four examples that intuitively demonstrate the effectiveness of the HASM. The deep-learning model initially detected landmarks as red stars; however, significant detection errors occurred in the X-ray images. In the (a), the lower left point of L3 was incorrectly detected, deviating from its ground-truth position. Similarly, in the (b), the detected position of the upper right point in L1 was considerably lower than its ground-truth position, because the region in the image was dark and the shape was ambiguous. Nevertheless, these errors were corrected by the proposed HASM as indicated by green stars, and the distance between the ground truth and detected positions decreased in both examples. In the case of (c), the presence of noise components such as white vertical lines and a black horizontal line made it difficult to analyze the image, and as a result, the upper right point of L5 was not correctly detected by the deep learning model. The (d) represents the case where interference from other organs made some of the vertebrae appear unclear in the image. This interference resulted in an initial error in the detection of the upper left point of L3. Similar to (a) and (b), the errors of these two cases were also reduced by applying HASM, and more reliable landmark detection results were obtained. HASM can integrate additional information on the statistical characteristics of landmark distribution into the image analysis results obtained from the deep-learning model. This advantage facilitates more robust landmark detection illustrated by the examples. However, as shown in Fig. 8, there are still cases where precise rectification is difficult, even when HASM is applied. In the (a), the vertebrae were not clearly visible in the image due to the influence of body tissues. In (b), the shape of L3 was abnormal. Consequently, the deep-learning model could not analyze the images correctly, and there were large errors in the initial landmark detection. Heatmap information could not be formed correctly because of the difficulty of image analysis in the deep-learning model. Relatively large errors remained, even after applying the HASM. Despite these limitations, the HASM still had merit and reduced errors to a certain extent. The large error in the white circle in (a) was decreased, causing the refined landmark to move closer to its correct position. In (b), the landmark for the lower-left point of L3 existed in R1 but not in R2 before correction. Using the HASM, the landmark could be relocated to R2, which was closer to its correct position. These two examples demonstrate the advantages and limitations of the HASM in handling errors caused by exceptional cases that are not included in the training data. When a deep-learning model fails to represent landmark location information in the heatmap results, accurate landmark detection using the HASM becomes limited. However, even when the locations of certain landmarks are challenging to determine, the HASM can estimate the likely locations of those landmarks based on the positions of other landmarks by utilizing the statistical distribution information of all landmarks. Therefore, although the HASM may not be able to correct all types of errors precisely, it can perform corrections to a generally acceptable distribution for errors exhibiting anomalous statistical distributions that fall outside the normal range.

**Table 6:** Data distribution for the case when assuming an error-free scenario in Pose-Net

Method	$\le E_{a,i}$	$\le 2E_{a,i}$	$\le 3E_{a,i}$	$\le 4E_{a,i}$	$\le 5E_{a,i}$	$>5E_{a,i}$
$wo\_HASM$	626	72	10	3	1	8
$w\_HASM$	646	63	4	1	2	4

**Table 7:** Performance indices of landmark detection when assuming an error-free scenario in Pose-Net

Method	$AE _{max}$ (mm)	$AE _{mean}$ (mm)	$NE _{max}$	$NE _{mean}$
$wo\_HASM$	6.1148	1.6174	0.8689	0.2299
$$w\_HASM$$	5.2378$$^{**}$$	1.6348	0.7429$$^{**}$$	0.2321

**p < 0.01 in paired samples *t*-test with $wo\_HASM$

### Influence of Deep-Learning Model Performance

**Fig. 9:**
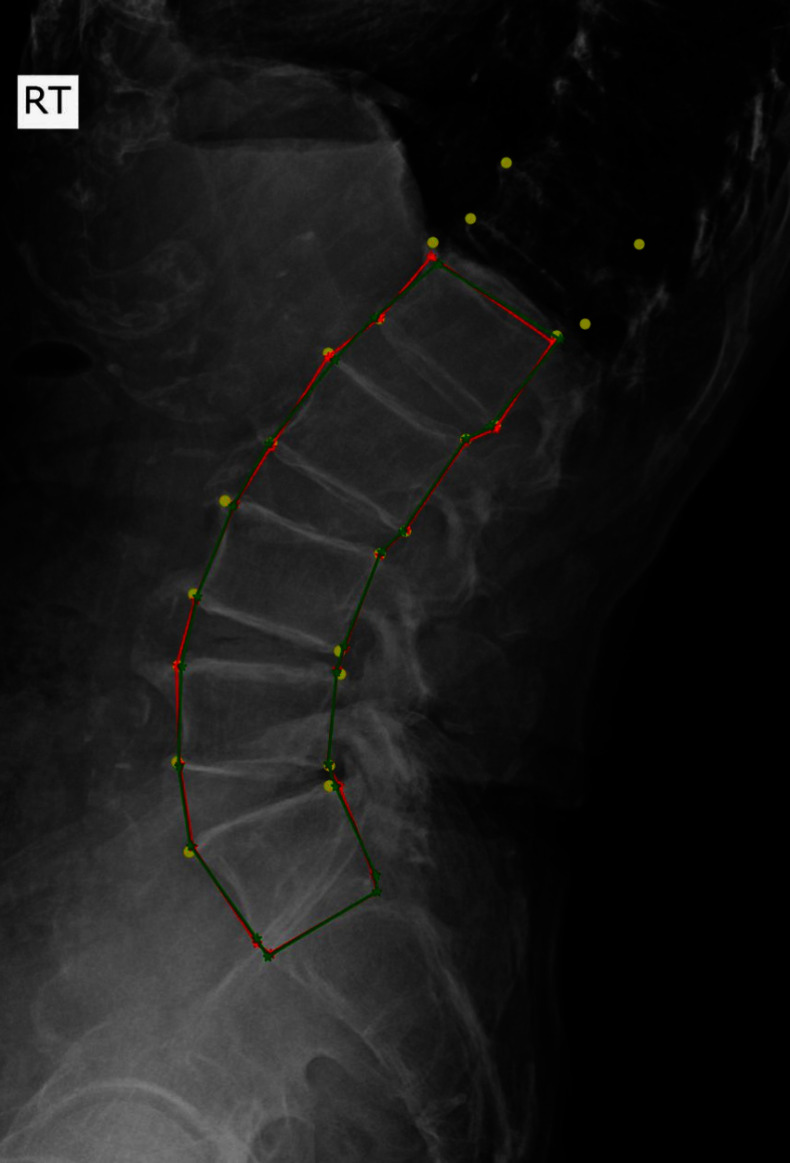
Example illustrating a case with large landmark detection errors owing to the failure of Pose-Net. The yellow circles are the ground-truth position of the landmarks. The red and green stars are detected and corrected landmarks, respectively

The proposed HASM was applied to the results of a deep-learning model, and the positions of the landmarks were corrected. Additional simulations were conducted to investigate the influence of the deep-learning model performance on the landmark detection results. This research utilized two deep-learning models for the two-step process: Pose-Net was used to identify the centers of the five lumbar vertebrae and the upper endplate line for S1, and M-Net was employed to analyze the cropped sub-images (Fig. 1). In the subsequent processes, inaccurate determination of the vertebral center in Pose-Net could lead to severe errors in landmark detection because the sub-images were cropped based on the detected center points. Indeed, significant errors in the interval [$>5E_{a,i}$] in Table 2 were observed owing to the failure of Pose-Net. Figure 9 shows an example in which Pose-Net failed to detect the correct center points. In this case, differentiating between L5 and S1 was difficult because of the lumbosacral transitional vertebrae [36]. Because there are varying opinions among physicians regarding the ground-truth position of S1, and HASM is not proposed to overcome this type of malpositioning, we exclude failure cases of Pose-Net in the following analysis. We investigated the effectiveness of the HASM by assuming an error-free scenario in Pose-Net. The ground-truth center positions of the vertebrae were used to crop the sub-images in the simulation. The landmark detection results of the simulation are presented in Tables 6 and 7. The landmark detection errors decreased overall compared with those in Tables 2 and 3 because of the absence of Pose-Net failures, and this change primarily affected the error cases in [$>5E_{a,i}$]. The advantages of the proposed HASM could also be identified in this scenario; both $AE _{max}$ and $NE _{max}$ exhibited a statistically significant decrease, and error reduction was observed across almost all intervals. The decreases in the $AE _{max}$ and $NE _{max}$ values after applying the HASM were 14.34% and 14.50%, respectively. These values were markedly larger than the values of 5.58% and 5.65% listed in Table 3. This was because the improved reliability of Pose-Net reduced the error cases that were difficult to correct owing to an incorrectly constructed sub-image, such as data in the interval $>5E_{a,i}$. The effectiveness of the proposed HASM became more apparent in this simulation because the proportion of correctable data using the HASM increased.

### Use of Post-Processing

The simulation results demonstrated that the proposed HASM could improve the robustness of landmark detection. However, there was a slight increase in $AE _{mean}$ and $NE _{mean}$ owing to subtle positional variations in the overall landmarks, as mentioned previously. These changes were much smaller than the reductions in $AE _{max}$ and $NE _{max}$. Nevertheless, an additional post-processing method can be implemented to mitigate the increase in $AE _{mean}$ and $NE _{mean}$ and to adjust the degree of positional changes from the HASM. A simple post-processing method involves moving the position of a detected landmark to the global maximum point of the heatmap when the distance between the two points is small. To define the criterion for proximity, $\tilde{E}_{a,i}$ can be calculated using Eqs. (6) to (10) with the detected landmark points instead of the ground-truth positions for each image. Then, $\gamma \tilde{E}_{a,i}$ can be used as the criterion. Tables 8 and 9 present the simulation results for various $\gamma$ values. The results show that, as $\gamma$ increased, $AE _{max}$ and $NE _{max}$ also increased, and the ratio of cases with a larger error than $E_{a,i}$ also expanded. However, the post-processing step allowed for a reduction in $AE _{mean}$ and $NE _{mean}$. For $\gamma \ge 0.05$, it was possible to achieve lower values of $AE _{mean}$ and $NE _{mean}$ compared to those of $wo\_HASM$ in Table 3, even with lower values of $AE _{max}$ and $NE _{max}$ and better data distribution results in Table 8. When $\gamma$ was less than 0.07, $AE _{max}$ and $NE _{max}$ remained statistically significantly smaller than $wo\_HASM$, and $AE _{mean}$ and $NE _{mean}$ also showed statistically significant decreases. As observed in the simulation results, the tradeoff between $AE _{max}$ and $AE _{mean}$ ($NE _{max}$ and $NE _{mean}$) can be controlled by employing the post-processing method. Although the post-processing was not utilized in other simulations in this study, because the focus was primarily on reducing $AE _{max}$ to enhance the robustness of landmark detection and the increase in $AE _{mean}$ was not substantial, it can provide users with options to customize the usage of the proposed HASM.

**Table 8:** Changes in data distribution according to $\gamma$ when applying post-processing to the HASM

$\gamma$	$\le E_{a,i}$	$\le 2E_{a,i}$	$\le 3E_{a,i}$	$\le 4E_{a,i}$	$\le 5E_{a,i}$	$>5E_{a,i}$
0.01	614	64	7	0	0	35
0.03	611	67	7	0	0	35
0.05	611	65	9	0	0	35
0.07	607	67	11	0	0	35
0.1	597	75	13	0	0	35

**Table 9:** Changes in performance indices of landmark detection according to $\gamma$ when applying post-processing to the HASM

$\gamma$	$AE _{max}$ (mm)	$AE _{mean}$ (mm)	$NE _{max}$	$NE _{mean}$
0.01	10.6276$$^{**}$$	3.1710$$^{**}$$	1.5131$$^{**}$$	0.4550$$^{**}$$
0.03	10.6344$$^{**}$$	3.1473	1.5142$$^{**}$$	0.4517
0.05	10.6977$$^{**}$$	3.1119	1.5235$$^{**}$$	0.4467
0.07	10.7626$$^{**}$$	3.1020$$^{*}$$	1.5324$$^{**}$$	0.4453$$^{*}$$
0.1	10.8185$$^{**}$$	3.1013$$^{*}$$	1.5401$$^{**}$$	0.4452$$^{*}$$

*p < 0.05 and **p < 0.01 in paired samples *t*-test with $wo\_HASM$ (Table 3)

**Table 10:** Data distribution for various methods to improve landmark detection

Method	$\le E_{a,i}$	$\le 2E_{a,i}$	$\le 3E_{a,i}$	$\le 4E_{a,i}$	$\le 5E_{a,i}$	$>5E_{a,i}$
Base	586	76	15	4	2	37
Base+CoordConv	592	78	11	2	0	37
Base+PAFs	601	67	13	1	1	37
Base+HASM	615	63	7	0	0	35
Base+CoordConv+PAFs	598	74	11	2	1	34
Base+CoordConv+PAFs+HASM	624	53	7	2	1	33

**Table 11:** Comparison results of performance indices of landmark detection

Method	$AE _{max}$ (mm)	$AE _{mean}$ (mm)	$NE _{max}$	$NE _{mean}$
Base	11.2491	3.1287	1.6026	0.4492
Base+CoordConv	11.0480	3.0951	1.5771	0.4447
Base+PAFs	11.0218	3.0886	1.5709	0.4436
Base+HASM	10.6209$$^{**}$$	3.1722$$^{**}$$	1.5121$$^{**}$$	0.4552$$^{**}$$
Base+CoordConv+PAFs	10.7717	3.0690	1.5353	0.4409
Base+CoordConv+PAFs+HASM	10.2739$^{*}$	3.1158	1.4628$^{*}$	0.4471

*p < 0.05 and **p < 0.01 in paired samples *t*-test with Base

### Comparison and Integration with Other Methods

As mentioned in the Introduction, various methods have previously been studied to enhance the robustness of landmark detection using lumbar X-ray images. The advantage of the proposed HASM is that it does not require any modification of the deep-learning network, unlike previous methods. Nevertheless, CoordConv and PAFs were selected from previous methods and applied to our deep-learning model because they required relatively minor modifications, such as altering some model layers and constructing additional target data for training. Subsequently, the landmark detection results for each method were simulated to compare the effectiveness of the HASM (Tables 10 and 11). In the tables, “Base” represents the performance of the deep-learning model itself for landmark detection. CoordConv and PAFs improved the overall performance. The number of data with $E_{max,i}$ no more than $E_{a,i}$ increased, and all performance indices were improved. These positive effects were more pronounced when CoordConv and PAFs were used together. However, $AE _{max}$ and $NE _{max}$ were lower when the HASM was used compared with CoordConv and PAFs, and the values were even better than when CoordConv and PAFs were combined. In addition, the decreases in $AE _{max}$ and $NE _{max}$ were statistically significant only when the HASM was employed. This was because of the increased robustness of landmark detection achieved using the HASM, and benefits could also be observed in the data distribution. Furthermore, the HASM could be applied in conjunction with CoordConv and PAFs without any network modification, resulting in a further reduction in $E_{max,i}$ compared to the CoordConv+PAFs case: $AE _{max}$ and $NE _{max}$ decreased by 4.62% and 4.72%, respectively, and the number of data belonging to the interval [$\le E_{a,i}$] increased by 26. The combined application of the HASM and CoordConv+PAFs also improved the four performance indices compared with using HASM alone. While the statistical significance was diminished for $AE _{max}$ and $NE _{max}$, the reductions in the two values were still statistically significant, with *p*-values of less than 0.05. The combined method led to a decrease in $AE _{mean}$ and $NE _{mean}$, which increased in Base+HASM relative to Base. The results in Tables 10 and 11 demonstrate the superior effectiveness of the proposed HASM for landmark detection compared with other methods. In addition, the proposed HASM can be combined with other methods to enhance the detection performance and increase stability further.

## Discussion

Deep-learning-based methods have frequently been utilized to analyze X-ray images and have recently achieved outstanding performance [7, 8]. However, as shown in Fig. 7, certain errors could still occur. Information on landmark distribution can help to alleviate these errors. CoordConv and PAFs were devised based on this idea. These methods provide guidance for deep-learning models to train their ability to utilize geometrical information. The proposed HASM shares a similar conceptual framework with these methods; the deep-learning model generates the analysis results of an image in the form of a heatmap, which is then refined by the shape model that contains the statistical information of the landmark distribution. The effectiveness of the HASM was validated in the experiments, and it outperformed CoordConv and PAFs. One reason for the superior results of the HASM is its effective utilization of the distribution characteristics of the entire landmark. Each lumbar vertebra exhibits a structural relationship. These regularities were incorporated into the shape model, which was then used to correct landmark positions. In contrast, the PAFs only employed the relationship information between two landmarks within the same vertebra, thereby limiting their ability to correct landmark positions. This resulted in reduced robustness to various error cases compared to the HASM. While a direct comparison was not conducted because of the substantial modifications required for the utilized deep-learning model, the PCA-based method [20] mentioned in the Introduction also has the same limitation. This method involves a two-step process similar to our study. In the first step, the center points of the whole vertebrae were determined by leveraging CNN models and PCA. However, in the second step, each vertebra was individually examined, and only the information on four landmarks within the same vertebra was used for the final landmark detection and correction. Compared to this method, our method used the distribution of whole landmark points. Another distinctive advantage of the HASM is its ability to adjust the incorporation of distribution information with the results of a deep-learning model via various parameters, including $n_P$ and $\mu$. This flexibility allows the HASM to utilize statistical information effectively based on the image quality or the ratio of abnormal vertebrae in the analysis data, leading to optimized performance. Other methods induce deep-learning models to learn statistical information; however, they lack the ability to control the influence of this information on the final result directly. In addition, CoordConv, PAFs, and the PCA-based method involve the training process of deep-learning models; however, the HASM can be applied as a post-processing technique independent of the model training or inference process. Consequently, the HASM can easily be applied to various models, and it is not necessary to retrain the deep-learning model to modify the HASM. In general, significant time and resources are required for model training, and numerous high-performance backbone models have been rapidly introduced. Therefore, this is a potential advantage of the proposed HASM. The differences between the proposed HASM and other methods introduced to increase robustness of vertebral landmark detection are summarized in Table 12.

**Table 12:** Comparative analysis of HASM and similar approaches using three standards: 1) utilization of the entire landmark distribution, 2) adjustable incorporation of statistical information, and 3) easy integration with other deep-learning techniques

Methods	Standard1	Standard2	Standard3
CoordConv [19]	$\bigcirc$	$\times$	$\times$
PAFs [18]	$\times$	$\times$	$\times$
PCA-based method [20]	$\bigtriangleup$	$\bigtriangleup$	$\times$
HASM	$\bigcirc$	$\bigcirc$	$\bigcirc$

The main contribution of this study is the proposal of a method that combines the ASM using statistical information with deep-learning techniques and its application to the landmark detection of lumbar vertebrae in X-ray images. Several studies have attempted to integrate the ASM with deep learning, albeit in different areas of application. In one approach, the results obtained from deep learning were post-processed using the ASM [24]. However, this was a simple combination of the two technologies, utilizing the deep-learning model output only as the initial value for the ASM. The correction process was identical to that of the conventional ASM. This limited the image analysis capability of the deep-learning model because its results were reinvestigated using traditional image analysis techniques. Other studies introduced methods that utilize deep-learning analysis results in the iterative refinement step of the ASM; however, the primary goal of most of these studies was image segmentation [25]. Therefore, unlike our approach, the deep-learning model in these studies produced a response map for the segmented regions rather than a Gaussian distribution heatmap for individual landmarks. In addition, other techniques were employed together to restrict the extent of the segmented regions in these methods. Another study demonstrated the applicability of a similar methodology to a landmark detection task [26]. This method proved the superiority of a deep-learning-based feature over traditional handcrafted features for the ASM. To achieve robust ASM parameter updates against outliers, generalized expectation maximization was employed instead of the gradient descent-based method used in the HASM, which considers Gaussian distribution heatmaps. Nonetheless, our study, which focused on lumbar vertebral landmarks, has several differences from other methods. As discussed in Section “Construction of Shape Model”, data imbalance in the lumbar vertebral images can introduce bias into the shape model. To address this challenge, this study augmented artificial spondylolisthesis data through simple geometric transformations. This feature improves the robustness of the landmark detection, as shown in Tables 2 and 3. To detect landmarks in high-resolution lumbar X-ray images, deep-learning models typically analyze sub-images corresponding to each landmark, generating heatmap results. Therefore, the HASM needed to investigate Gaussian distribution-shape heatmaps from individual sub-images for each landmark, rather than a single image. This method updates the overall landmarks by aggregating information from the sub-images. The HASM can enhance landmark detection robustness, but may introduce subtle positional variations. This study also showed that post-processing can be employed to address this problem, as discussed in Section “Use of Post-Processing”.

Despite the many advantages of the proposed method, there are limitations and challenges that must be overcome. While the proposed method can improve the robustness of landmark detection, its correction performance may be limited in cases in which the deep-learning model cannot generate heatmaps that provide sufficient information on landmark location owing to noise and morphological abnormalities of the vertebrae, as shown in Fig. 8. This implies that the performance of the deep-learning model must be improved through various network architectures and learning techniques to realize the potential of the HASM fully. The development of a deep-learning model with enhanced image analysis performance remains challenging. As the algorithm complexity was not thoroughly investigated in this study, we are also considering research on model optimization, in terms of not only performance but also computational efficiency. Another limitation of the HASM is the presence of subtle positional variations in the overall landmarks, although these changes are insignificant. Post-processing can mitigate these positional variations, but may potentially limit the chance of reducing the maximum error in each image. Therefore, finding a new approach to suppress positional variations without compromising the effect of maximum error reduction is a key focus for future research. In addition, we plan to develop more diverse application algorithms, such as integrating the results from multiple shape models or assigning different weights to individual landmarks when applying the shape model.

## Conclusions

HASM was proposed in this paper to improve the robustness of landmark detection for lateral X-ray images of lumbar vertebrae. HASM is an iterative process that revises the initial positions of detected landmarks by utilizing both the statistical characteristics of landmark distribution and the image analysis results of a deep-learning model. The proposed method can effectively use information from the entire landmark distribution and adjust the influence of statistical information on the final results, unlike other methods with similar objectives. In addition, the HASM, which is specifically designed for lumbar X-ray image analysis, distinguishes itself from other methodologies that combine the ASM and deep learning through the use of artificial spondylolisthesis data, heatmap information of multiple sub-images, and the post-processing. The effectiveness of the HASM was verified through various simulations, demonstrating its ability to reduce large detection errors and increase the stability in identifying vertebral landmarks. Furthermore, one of its major advantages is its broad compatibility. The HASM can easily be integrated with a deep-learning model that provides heatmap results for each landmark, as discussed in Section “Comparison and Integration with Other Methods”. This enables the HASM to synergize with other deep-learning-based techniques, further enhancing the robustness and effectiveness of landmark detection. The improved landmark detection performance is beneficial for automating X-ray image interpretation in the medical field (Supplementary Materials 3 and 4). Automated image diagnosis can advance healthcare systems by increasing efficiency, reducing healthcare costs, and benefiting both medical professionals and patients.

## Supplementary Information

Below is the link to the electronic supplementary material.

Supplementary file 1 (pdf 235 KB)

## Data Availability

Some part of the data used for this research is available at https://services.informatics.buu.ac.th/spine/, and the entire data will be released soon at the site.
