# Supplementary material for: Heatmap-Based Active Shape Model for Landmark Detection in Lumbar X-ray Images
Source: J Imaging Inform Med. 2024 Aug 5;38(1):291–308. doi: 10.1007/s10278-024-01210-x (PMC11811376; doi:10.1007/s10278-024-01210-x)
Supplement: Supplementary file 1 — (pdf 235 KB) [file 10278_2024_1210_MOESM1_ESM.pdf]

# 1 Number of iterations for HASM

The HASM corrects landmarks through a two-step iterative process. The iteration process is performed a predetermined number of times or terminated early when the changes in landmark positions are not significant. The number of iterations should be sufficiently large for the algorithm results to converge. However, a large number of iterations increases the computation time. We compared the performance for different numbers of iterations and confirmed that the algorithm results converged correctly when the number of iterations was 30 or more. Therefore, the number of iterations was set to 30 in this study.

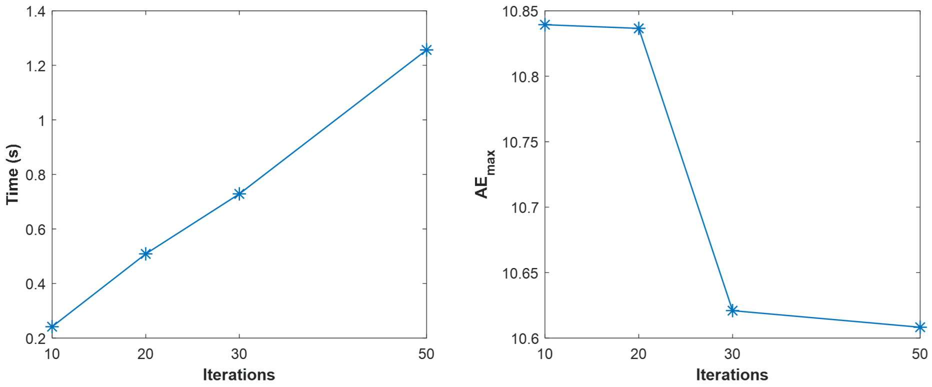


**Fig. 1** Average computation time per test image (left) and landmark correction performance (right) for different iteration numbers

The computation times when the number of iterations was set to 30 are listed in Table 1. The HASM used in this study is designed to operate in a CPU. There is room for improvement in terms of the computation time by using GPUs and other optimization techniques.

**Table 1** Average computation time for a test image in each stage

| Stage | | Deep learning model (Pose-Net + M-Net) | HASM |
|---|---|---|---|
| Time (s) | CPU | 1.5900 | 0.7287 |
| | GPU | 0.6226 | n.a. |

# 2 Selection of $n_P$ and $\mu$

The HASM uses $n_P$ and $\mu$ to control the degree to which the information of the shape model is incorporated into the landmark location correction. These two parameters are designated using a grid-search method. Validation data are tested on predefined grid points for the parameters, and the two values corresponding to the case with the highest performance (the lowest $NE_{max}$) are selected as the final $n_P$ and $\mu$. The following tables present the simulation results obtained to determine the parameters. The performance corresponding to each grid point is represented by $NE_{max}$.

## 2.1 Base+HASM

**Table 2** Validation data

| $\mu$ \ $n_P$ | 20 | 21 | 22 | 23 |
|---|---|---|---|---|
| 0.01 | 1.0846 | 1.0859 | 1.0933 | 1.1528 |
| 0.015 | 1.0826 | 1.087 | 1.0912 | 1.1534 |
| 0.02 | **1.0802** | 1.0875 | 1.0895 | 1.1531 |
| 0.025 | 1.0811 | 1.0852 | 1.0925 | 1.1562 |
| 0.03 | 1.0808 | 1.0854 | 1.0901 | 1.1552 |
| 0.035 | 1.0822 | 1.0848 | 1.0909 | 1.1553 |

**Table 3** Test data

| $\mu$ \ $n_P$ | 20 | 21 | 22 | 23 |
|---|---|---|---|---|
| 0.01 | 1.5451 | 1.5180 | 1.5199 | 1.5532 |
| 0.015 | 1.5411 | 1.5157 | 1.5181 | 1.5207 |
| 0.02 | 1.5121 | 1.5109 | 1.5134 | 1.5470 |
| 0.025 | 1.5396 | 1.5092 | 1.5136 | 1.5137 |
| 0.03 | 1.5114 | 1.5092 | 1.5094 | 1.5123 |
| 0.035 | 1.5382 | 1.5097 | 1.5424 | 1.5108 |

## 2.2 Base+CoordConv+PAFs+HASM

**Table 4** Validation data

| $\mu$ \ $n_P$ | 20 | 21 | 22 | 23 |
|---|---|---|---|---|
| 0.01 | 1.0804 | 1.0783 | 1.0764 | 1.0833 |
| 0.015 | 1.0769 | 1.0768 | 1.0766 | 1.0794 |
| 0.02 | 1.0780 | **1.0744** | 1.0760 | 1.0752 |
| 0.025 | 1.0786 | 1.0785 | 1.0772 | 1.0774 |
| 0.03 | 1.0777 | 1.0773 | 1.0788 | 1.0773 |
| 0.035 | 1.0784 | 1.0816 | 1.0806 | 1.0769 |

**Table 5** Test data

| $\mu$ \ $n_P$ | 20 | 21 | 22 | 23 |
|---|---|---|---|---|
| 0.01 | 1.4969 | 1.4640 | 1.4651 | 1.4630 |
| 0.015 | 1.4930 | 1.4641 | 1.4589 | 1.4594 |
| 0.02 | 1.4918 | 1.4628 | 1.4567 | 1.4574 |
| 0.025 | 1.4956 | 1.4628 | 1.4556 | 1.4564 |
| 0.03 | 1.4929 | 1.4617 | 1.4576 | 1.4560 |
| 0.035 | 1.4912 | 1.4621 | 1.4574 | 1.4837 |

# 3 Clinical assessment parameters of utilized dataset

Clinical assessment parameters, including the lumbar lordotic angle, wedge angle of L4/L5, and lumbosacral angle were calculated from the 3,600 X-ray images, and the results are presented in this section [S1]. This information can provide insights into the structural characteristics and morphological diversity of the utilized lumbar spine data.

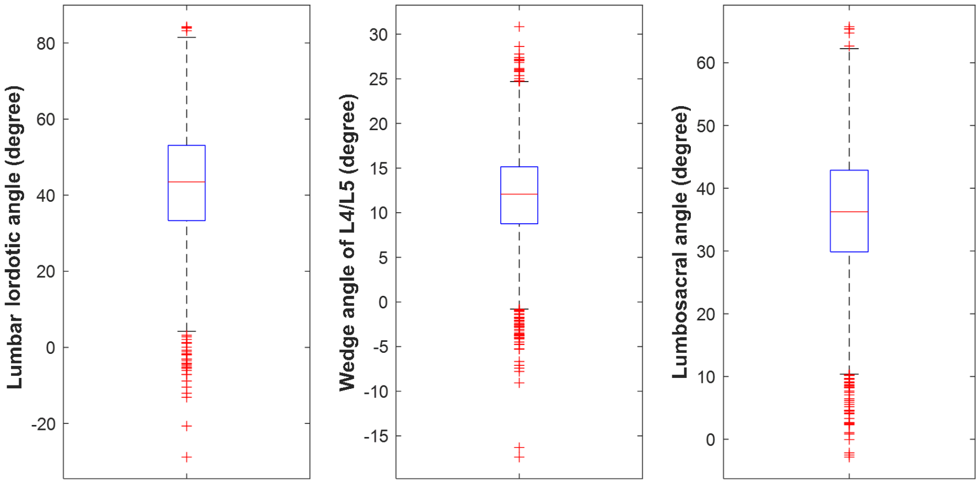


**Fig. 2** Boxplots to visualize the clinical assessment parameters. The red lines and cross marks represent the median values and outliers, respectively. The blue boxes indicate data between the 25th and 75th percentiles


[S1] Kocyigit et. al., Comparison of lumbosacral alignment in geriatric and non-geriatric patients suffering low back pain, Pakistan Journal of Medical Sciences, 2018.

# 4 Estimation of clinical assessment parameters using HASM

This section presents the results of the change in clinical parameter estimation when utilizing the HASM. The results were obtained by applying the proposed HASM without any modifications (Section 3.3), and detailed adjustments may be required to improve its performance.

**Table 6** Mean (standard deviation) of absolute errors for estimated clinical assessment parameters

| Parameters | *wo_HASM* | *w_HASM* |
|---|---|---|
| Lumbar lordotic angle | 4.4390 (5.6233) | 4.2770 (4.8103) |
| Wedge angle of L4/L5 | 2.5447 (3.3224) | 2.5116 (2.3477) |
| Lumbosacral angle | 3.0741 (4.3856) | 3.0317 (4.3117) |

The degree of vertebral slippage can be represented by the P-grade, which assesses the misalignment between the upper and lower vertebrae. The P-grade for L5 to S1 was calculated based on the detected landmarks using a simple method [S2], and cases with a value >20% were considered an abnormality. The HASM could improve the classification accuracy for normal and abnormal cases from 97.6389% to 98.4722% by reducing the number of cases in which the landmark detection error was large.

**Table 7** P-grade estimation results where abnormal cases were considered as positive

| Method | True positive | True negative | False positive | False negative | Accuracy (%) |
|---|---|---|---|---|---|
| *wo_HASM* | 2 | 701 | 8 | 9 | 97.6389 |
| *w_HASM* | 3 | 706 | 3 | 8 | 98.4722 |

[S2] Trinh et al. Detection of lumbar spondylolisthesis from X-ray images using deep learning network, Journal of Clinical Medicine, 2022.
